# Molecular cytological analysis of alien introgressions in common wheat lines derived from the cross of *TRITICUM AESTIVUM* with *T. kiharae*

**DOI:** 10.1186/s12870-020-02407-2

**Published:** 2020-10-14

**Authors:** Оlga Orlovskaya, Nadezhda Dubovets, Lylia Solovey, Irina Leonova

**Affiliations:** 1 Institute of Genetics and Cytology of the National Academy of Sciences of Belarus, Akademycheskaya Street 27, 220072 Minsk, Belarus; 2 The Federal Research Center “Institute of Cytology and Genetics of Siberian Branch of the Russian Academy of Sciences”, Prospekt Lavrentyeva 10, Novosibirsk, Russian Federation 630090

**Keywords:** Common wheat *T. aestivum* L., Synthetic wheat *T. kiharae*, Introgression lines, C-banding, SSR analysis, Microsporogenesis, Cytological stability

## Abstract

**Background:**

*Triticum kiharae* (A^t^A^t^GGDD, 2n = 42) is of interest for the improvement of bread wheat as a source of high grain protein and gluten content, as well as resistance to many diseases. The use of *T. kiharae* for the improvement of *T. aestivum* L. is complicated by the fact that the homology degree of their genomes is low and this leads to an unbalanced set of chromosomes in the gametes of its first generations and the elimination of some genotypes. The aim of this study was to analyze the nature of alien introgressions and their effect on the cytological stability of hybrids obtained from crossing of bread wheat varieties with *T. kiharae*.

**Results:**

Using C-banding, the presence of entire chromosomes of *T. kiharae* in the karyotypes of hybrid lines (intergenomic substitution 2G/2B), chromosome arms (centric translocation Т2A^t^S:2AL) and large inserts in the form of terminal translocations involving chromosomes of 1st, 3rd and 5th homoeologous groups of B- and G-genomes were found. Molecular markers revealed short introgression of *T. kiharae* into the genome of common wheat varieties. The highest introgression frequency was shown for 1A, 1B, 2A, 5B, and 6A chromosomes, while no foreign chromatin was detected in 4A and 4B chromosomes. A high level of cytological stability (a meiotic index of 88.18–93.0%) was noted for the majority of introgression lines. An exception was found for the lines containing the structural reorganization of chromosome 5B, affecting the main genes of chromosome synapsis in terms of their functioning.

**Conclusions:**

During the stabilization of hybrid karyotypes, the introgression of genetic material from *T. kiharae* into the genome of *T. aestivum* occurs in the form of short fragments detectable only by molecular markers and in the form of whole chromosomes (intergenomic substitution) and their large fragments (centric and terminal translocations). The level of cytological stability achieved in F_10_ by the majority of introgression lines ensures the formation of functional gametes sufficient for the successful reproduction of the obtained hybrids.

## Background

*Triticum kiharae* Dorof. et Migusch. (A^t^A^t^GGDD, 2n = 42) is an artificially synthesized amphidiploid developed in the 60s of the twentieth century at Kyoto University (Japan) by crossing of *T. timopheevii* Zhuk. (A^t^A^t^GG, 2n = 28) and *Aegilops tauschii* Coss. (DD, 2n = 14) [1]. It combines a set of features of both parental species, as positive – resistance to most diseases and pests, high grain protein and gluten content, the presence of flour substances with antimicrobial properties – defensins [2, 3], and negative – low grain yield, hard threshing kernels, very strong gluten [1]. It is known that *T. timopheevii* Zhuk. and related species are a separate branch of polyploid wheat evolution and contain complete sets of chromosomes of A^t^ - and G-genomes, which differ in the karyotype structure and C-banding from the chromosomes of A- and B-genomes of common wheat [4, 5].

The use of *T. kiharae* for the improvement of *T. aestivum* is complicated by the fact that the degree of chromosome homology between their genomes is low that results in the imbalance of chromosome sets in the gametes of F_1_ hybrids and subsequent generations and leads to low fertility. The consequence of this is the elimination of a number of genotypes in generations that narrows their hereditary diversity range and possibility to select valuable forms with different gene combinations.

Monitoring the process of alien chromatin introgression into the common wheat genome is a key to success in efforts to enrich the gene pool with economically important genes from related species. One of the main methods used for the localization of alien introgression is C-banding, which allows to detect the substitution of chromosomes or their arms, as well as extended translocations and large deletions [6] according to the pattern of the eu- and heterochromatin blocks in each chromosome. However, many cereal genomes contain a small amount of heterochromatin, which limits the use of this method.

With the construction of saturated molecular genetic maps of bread wheat and other cereal species, it became possible to use molecular markers to detect structural changes in low-heterochromatic genomes and identify short introgression fragments. However, this method also has limitations, because it does not identify non-homologous rearrangements and translocations [7]. We have shown earlier [8, 9] that only the combination of these two methods makes it possible to reveal the nature of structural transformations arising in the course of genome stabilization of remote hybrids. This investigation provides an assessment of the genomic composition of hybrid lines performed using both approaches. The aim of the study was to analyze the nature of alien introgressions and their impact on the cytological stability of hybrids obtained from the cross of bread wheat varieties with *T. kiharae*.

## Results

### Development of wheat lines with *T. kiharae* genetic material

As a result of hybridization of bread wheat varieties *T. aestivum* L. (AABBDD, 2n = 42) with *T. kiharae* (A^t^A^t^GGDD, 2n = 42), hybrid grains were obtained in six cross combinations (two of them are direct, where *T. kiharae* was used as a mother plant, and 4 – inverse, where synthetic wheat served as a pollinator). Influence of the parental wheat variety and the direction of crossing on the success of interspecific hybridization should be noted. Thus, the highest percentage of fertility was found in cross combinations with the Saratovskaya 29 variety, both in its direct and reverse hybridizations. At the same time, the grain formation was much higher (47.92–53.85%) in the direct cross combination (*T. kiharae*×Festivalnaya, *T. kiharae*×Saratovskaya 29) than in reverse directions − 1.79-9.09%.

We detected the inheritance of morphological features typical to *T. kiharae* in the F_1_ generation, and a high level of the reorganization process in F_2–4_ generations [10]. Among F_4_ progenies, hybrid forms were identified, containing morphological traits inherited from *T. kiharae* (absence of a wax cover on the leaf, stem and spike; purple coloring of a stem; dark color and pubescence of glumes, a speltoid spike). These offsprings were included in further studies. For the molecular cytogenetic analysis, we used seven hybrid lines selected on the basis of inheritance of morphological characteristics and productivity in generations F_4_ - F_9_.

### C-banding

As already noted, the C-banding technique effectiveness directly depends on the saturation of a differential staining pattern of chromosomes, within which structural transformations are analyzed. If these reorganizations occur in the chromosome regions, which do not contain diagnostic heterochromatin bands, it is not possible to identify rearrangements using C-banding. This is a reason that most of the chromosomal aberrations we have identified affect the chromosomes of B- and G wheat genomes, which are characterized by a high heterochromatin content.

**Fig. 1:**
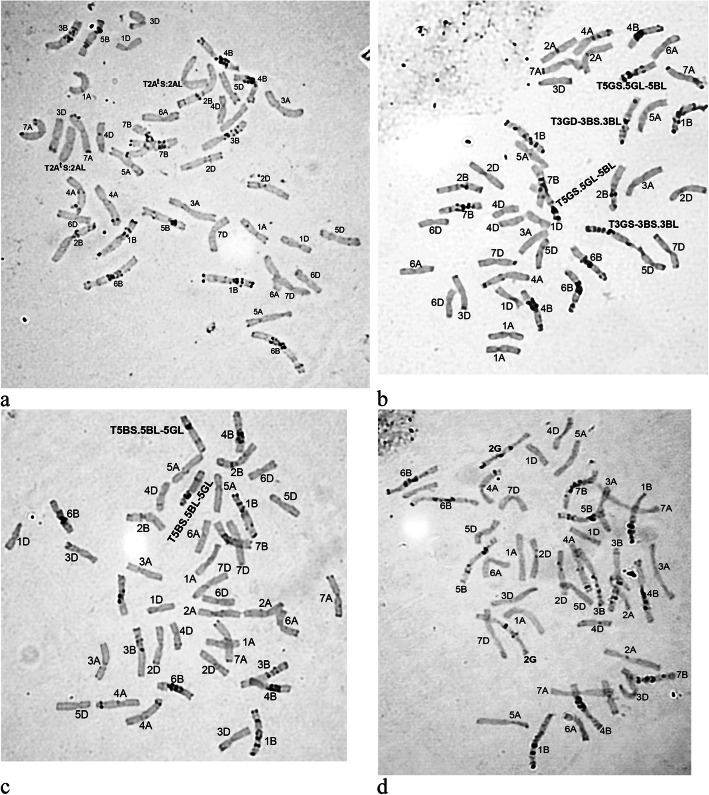
Karyotypes of common wheat lines (F_10_) with various introgressions of *T. kiharae* genetic material (С-banding). **а** – line 19, **b** – line 25–2, **c** – line 34–1, **d** – line 28

As for the introgression of the genetic material of the A^t^ genome of *T. kiharae*, C-banding revealed only one variant of such event – centric T2A^t^S.2AL translocation, which is present in the disomic state in all plants of line 19 (Fig. 1a). Such translocations result from the centromere misdivision of univalent chromosomes during their anaphase moving in meiosis with the formation of telocentrics, further random fusion of which leads to the formation of recombinant chromosomes [11].

In the studied lines, C-banding did not reveal the introgression of a *T. kiharae* D-genome genetic material into the genome of common wheat varieties.

The transfer of the *T. kiharae* G-genome genetic material was carried out mainly by means of translocations with breakpoints in interstitial chromosome regions that were formed between the corresponding homoeologous G- and the orthologous to it B-genome. In total, four types of such translocations involving 1st, 3rd and 5th- homoeologous group chromosomes were found in the studied lines.

Thus, in line 20–1, the aberrant T1BS.1BL-1GL chromosome was detected in the disomic state, resulting from the terminal part transfer of a long arm of chromosome 1G to the distal part of a long arm of chromosome 1B. In the karyotypes of line 25–2, two translocations were identified: T3GS-3BS.3BL and T5GS.5GL-5BL (Fig. 1b). In the first case, the terminal fragment of a short arm of chromosome 3G was transferred to the short arm of chromosome 3B. In the second case, the modification affected the long arm of chromosome 5G, to the distal part of which the terminal fragment of a long arm of chromosome 5B was transferred.

Chromosome 5G was also involved in the structural rearrangements that occurred during the karyotypes’ stabilization of lines 34–1 and 34–2. However, the translocation identified here is reciprocal to the translocation described for line 25–2: the terminal fragment of a long arm of chromosome 5G was transferred to the distal part of 5B chromosome (Fig. 1c).

In addition to the introgression of *T. kiharae* chromatin in the form of a chromosome arm or large chromosome segments, C-banding revealed the formation of an intergenomic chromosome substitution: in the karyotypes of line 28, a pair of 2G chromosomes substituted a pair of 2B chromosomes (Fig. 1d). A high frequency of the similar substitution in offsprings obtained from the crossing of common wheat varieties with *T. kiharae* and *T. timopheevii* was noted earlier by a number of authors [5, 7, 12, 13]. The only line that did not demonstrate any changes in the C-banding pattern when compared to the original wheat variety is line 31, probably due to the different direction of crossing (the wheat variety in this case served as a mother component of hybridization).

### SSR-analysis

Molecular analysis of introgression lines was carried out using 82 SSR markers and most of them revealed polymorphism between parental wheat varieties and *T. kiharae* (Additional file 1). The analysis of PCR spectra showed that 27% of markers are not synthesized fragments in the *T. kiharae* genome. According to genotyping results, 241 alleles were amplified with introgression lines’ DNA (2.9 alleles per locus on average), which was lower than in wheat varieties (271 alleles, 3.3 alleles per locus). Indices of genetic diversity were also higher in parental varieties (0.62) as compared to introgression lines (0.54) due to the presence of genetic material *T. kiharae* in the genome of lines.

**Fig. 2:**
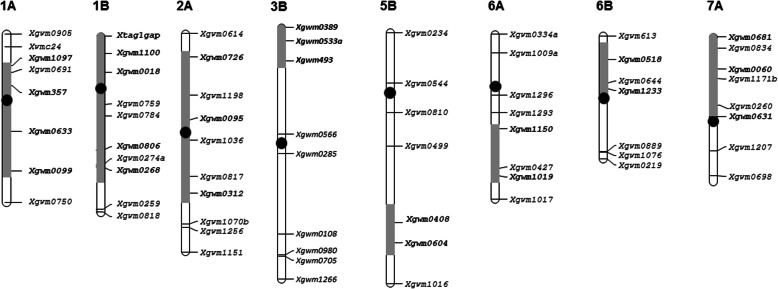
Schematic illustration of *T. kiharae* genome fragments for line 19. The order of microsatellite markers corresponds to the genetic maps of *T. aestivum* chromosomes (Röder et al., 1998). Dark blocks indicate the length of the introgression fragments. Markers colored in dark amplify fragments specific for the *T. kiharae* genome

Comparative analysis of the amplification spectra of SSR markers between *T. kiharae*, wheat varieties and introgression lines showed differences in the number and chromosomal localization of *T. kiharae* fragments in the genome of introgression lines. The number of introgression fragments varied from four to eight, with the greatest number noted in line 19 (Table 1; Fig. 2). No SSR alleles specific to *T. kiharae* were found in line 31, which is consistent with C-banding results.

**Table 1:** Chromosomal localization of *T. kiharae* genome fragments in introgression lines according to SSR analysis

Line	Chromosome										
19	1А	1В	2A	–	3BS	–	5BL	6AL	6BS	7AS	–
20–1	1А	1BL	–	–	–	5AL	5BL	–	–	7AS	–
25–2	1А	1BS	2AL	–	3BS	–	5BL	6AL	–	–	–
28	1А	1B	2А	2BL	–	5AL	–	–	–	–	–
34–1	1А	–	–	–	–	–	5BL	6AL	–	–	7BL

According to microsatellite analysis, no introgressions in D genome chromosomes were detected in hybrid lines. We do not exclude the presence of *Ae. tauschii* short ragments in D genome chromosomes. However, to identify such introgressions, it is necessary to use an additional number of SSR markers. Introgressions were found in A genome: chromosome 1A (all studied lines), 2A (lines 19, 25–2 and 28), 5AL (lines 20–1 and 28), 6AL (19, 25–2 and 34–1) and 7AS (19, 20–1). Using C-banding, structural changes were detected only in the chromosome 2A of line 19. The presence of rearrangements in other chromosomes of A genome was not established due to the small number of diagnostic heterochromatin blocks in chromosomes A^t^ of *T. kiharae*, which was also shown earlier by Badaeva et al. [5, 13].

Amplification of PCR fragments specific to *T. kiharae* was detected using SSR markers localized in the short arm of chromosome 3B (lines 19 and 25–2). Single introgressions in chromosomes 2BL, 6BS and 7BL were found only in lines 28, 19 and 34–1, respectively (Table 1). Comparison of the alien chromatin size showed that the lines differ in the length of introgressed fragments in chromosomes 1A, 1B and 2A. In lines 20–1, 28 and 34–1, the introgression in chromosome 1A captured a long chromosome arm and part of its short arm. The alien fragment in line 25–2 was the same length as in line 19 (Fig. 2), but significantly shorter than in other lines. Lines 19 and 28 were similar in the length of introgression fragments in the chromosome 1B. In line 20–1, a long arm of chromosome 1B was replaced, while the short arm substitution was found in line 25–2.

Differences in the length of introgression fragments were also detected in chromosome 2A: in line 19, a short arm of the chromosome was replaced; in line 25–2 – its long arm; in line 28, alien fragments were noted in both chromosome arms. As for the length of introgressed fragments in chromosomes 3B, 5A and 6A, no differences between the lines were found according to the SSR analysis. It should be noted that in lines 20–1 and 28, there seems to be a complete long arm replacement of chromosome 5A with a long arm of 5A^t^ from *T. kiharae*.

All lines, except for line 28, showed changes in the long arm of 5B chromosome. In lines 19 and 20–1, only two SSR markers of the telomeric 5BL region amplified a fragment typical of *T. kiharae* (Fig. 2), which indicates the presence of short alien fragments and inability to identify them by C-banding. For lines with large *T. kiharae* chromatin introgression (25–2 and 34–1), microsatellite analysis data coincide with C-banding results.

### Microsporogenesis

Study of meiosis in pollen mother cells (PMC) has shown the high cytological stability of F_10_ lines. At metaphase I, the average number of univalents per microsporocytes varied depending on the wheat variety from 0 to 0.07 (Table 2). In *T. kiharae*, this parameter was slightly higher and amounted to 0.4, but was within the norm noted for bread wheat (5%).

**Table 2:** The average frequencies of various chromosomal associations in metaphase I of meiosis in common wheat lines F_10_ with introgression of *T. kiharae* genetic material and their parental forms

Genotype	Line	Bivalents			Univalents
		closed	opened	in total	
Rassvet×*T.kiharae*	31	20.0 ± 0.18	0.96 ± 0.18	20.97 ± 0.03	0.07 ± 0.06
*T. kiharae*×Saratovskaya 29	19	19.53 ± 0.19	1.33 ± 0.19	20.9 ± 0.06	0.2 ± 0.11
	20–1	19.13 ± 0.27	1.8 ± 0.25	20.93 ± 0.05	0.13 ± 0.09
	25–2	19.1 ± 0.31	1.67 ± 0.26	20.77 ± 0.10	0.47 ± 0.21
*T.kiharae*×Festivalnaya	28	19.7 ± 0.21	1.23 ± 0.20	20.93 ± 0.05	0.13 ± 0.09
	34–1	17.83 ± 0.21	2.87 ± 0.18	20.7 ± 0.09	0.6 ± 0.19
	34–2	17.73 ± 0.28	2.4 ± 0.31	20.13 ± 0.12	1.8 ± 0.26
Rassvet		20.03 ± 0.16	0.97 ± 0.16	21.0 ± 0	0
Saratovskaya 29		19.6 ± 0.18	1.37 ± 0.19	20.97 ± 0.03	0.07 ± 0.06
Festivalnaya		19.5 ± 0.21	1.43 ± 0.21	20.97 ± 0.03	0.06 ± 0.06
*T.kiharae*		18.53 ± 0.27	2.27 ± 0.25	20.8 ± 0.09	0.4 ± 0.18

A high level of bivalent chromosome pairing at metaphase I was also found in all hybrid lines (Table 2). It should be noted that in the lines from the cross combinations of *T. kiharae*×Festivalnaya and *T. kiharae*×Saratovskaya 29, a sufficiently high level of chromosome synapsis was already established in F_2_ hybrids [14]. The number of chromosomes included in the bivalents of those hybrids was at the level of 90.8–96.2%. As a rule, 2 or 4 chromosomes did not participate in the process of mating. The maximum number of univalents identified in F_2_ was eight.

Analysis of metaphase I in F_10_ lines showed that the number of chromosomes forming a bivalent in most lines is close to 100%. The highest level of synapsis similar to the original wheat variety was found in line 31 characterized by the absence of foreign introgressions. In lines 19, 20–1 and 28, single PMC with two univalents occurred; line 25–2 had 60% of cells with two univalents among a relatively small number of PMC with disorders (16% of the analyzed PMC) and the remaining 40% – with four. The greatest number of PMC with disorders at that stage of meiosis (73.3%) and the maximum number of univalents (6–3.3% of PMC) was found in line 34–2.

Completing the analysis of metaphase I, it should be noted that during the change of generations there is a significant reduction in the number of open bivalents: in the F_2_ progeny, their average number ranged from 4.0 to 5.4 [14], in F_10_ – from 0.96 to 2.87 (Table 2). Despite a rather high level of chromosome pairing in F_2_, the subsequent stages of meiosis in that generation proceeded with significant disorders, resulting in the low percentage of normal tetrad (the so-called meiotic index, which is an indicator of the normal type of meiosis) varied from 0 to 32% [14]. The results obtained in this study indicate that up to the tenth generation there was a significant stabilization of the meiotic cycle (Table 3). The percentage of normal tetrad in F_10_ lines was at a high level and exceeded 90% as a rule. The main abnormality at that stage of meiosis was the presence of micronuclei, the number of which varied in the analyzed material from 1 to 6, but most often formed a tetrad with one and two micronuclei. Pollen mother cells containing six micronuclei were noted only in two lines 34–1 and 34–2 of the *T. kiharae* × Festivalnaya cross combination. Their frequency was very low and at the level of 0.91–1.67% of the total number of analyzed cells.

**Table 3:** Characteristics of meiosis in common wheat lines F_10_ with introgression of *T. kiharae* genetic material and their parental forms

Genotype	Line	PMC without aberrations, %			Meiotic index,%
		anaphaseI	metaphase II	anaphaseII	
Rassvet×*T.kiharae*	31	76.25	88.75	92.5	92.0
*T. kiharae*×Saratovskaya 29	19	54.67	80.65	88.0	93.0
	20–1	82.86	86.25	78.57	92.0
	25–2	85.0	80.0	77.5	90.91
*T.kiharae*×Festivalnaya	28	62.9	90.67	91.66	88.18
	34–1	76.0	63.75	71.11	77.0
	34–2	72.5	44.29	48.75	55.83
Rassvet		80.0	96.67	91.12	99.0
Saratovskaya 29		82.86	91.25	96.36	99.0
Festivalnaya		82.5	90.91	88.32	84.55
*T.kiharae*		74.0	74.29	61.43	80.0

In general, the analysis of microsporogenesis allows us to conclude that the level of cytological stability achieved in F_10_ by the majority of introgression lines ensures the formation of functional gametes in the amount sufficient for the successful reproduction of lines.

## Discussion

This study presents investigation results of cytological stability and chromosomal rearrangements in common wheat introgression lines obtained from the crosses with synthetic wheat *T. kiharae*. According to the literature, the fertility of the first and subsequent generations of hybrids obtained as a result of interspecific hybridization largely depends on the genetic background of bread wheat. Thus, the comparison of a number of kernels in interspecific hybrids from crosses between bread wheat cultivars Skala, Novosibirskaya 67, Botanicheskaya 2, Altaiskaya 81, Ghnitsa and Chinese spring with *T. timopheevii* (genome A^t^A^t^GG) showed that fertility was significantly higher in the hybrids derived on the base of Novosibirskaya 67 and Skala [15]. However, a ploidy level of the recipient has no significant effect on the fertility and chromosome pairing of interspecific hybrids [16–19].

It is also known that the direction of hybridization influences the fertility and cytological stability of interspecific hybrids significantly. Thus, in hybridization experiments between genus *Triticum* species, it was shown that if common wheat is used as female and diploid wheat as a pollinator, the fertility is much lower than in reverse combinations [20–22]. Analysis of crossing results of hexaploid (2n = 42) and tetraploid (2n = 28) wheat obtained earlier evidenced that fertility is much higher when the pollinator is a multichromosomal species [14]. For example, in the combination, where *T. persicum* was crossed as a mother plant with the bread wheat cultivar Toma, the fertility index was three times higher as compared with the reverse combination.

Molecular cytological analysis of *T. aestivum/T. kiharae* introgression lines indicate a sufficiently high frequency of *T. kiharae* genetic material introgression into the genome of common wheat varieties with clear predominance of small fragments detected by molecular markers. Using the C-banding method, the presence of entire chromosomes of *T. kiharae* (intergenomic substitution 2G/2B) and chromosome arms (centric translocation Т2A^t^S:2AL) was found. However, the predominant method used for the G-genome chromatin transfer was the formation of terminal translocations, leading to the formation of recombinant chromosome arms, containing genetic material of both wheat varieties. Taking into account the character of identified translocations, it is logical to assume that the mechanism of their formation is crossover exchanges of chromosome segments during homoeologous pairing in early generations. Significantly favorable conditions for such an exchange existed in F_1,_ where all chromosomes of genomes A, A^t^, B and G were in a monosomal state, and a *Ph1* locus was present in one dose. This assumption is supported by the presence of reciprocal translocations of T5GS.5GL-5BL in the analyzed lines (line 25–2 *T. kiharae*×Saratovskaya 29) and T5BS.5BL-5GL (lines 34–1 and 34–2 *T. kiharae*×Festivalnaya). It is important to note that from two products of chromosome reciprocal exchanges in different combinations of crossing there was the selection of different variants of recombinant chromosomes. This fact indicates the influence of the genetic background of the hybrid form (in this case, the original wheat variety) on the introgression process of an alien genetic material.

Identified aberrant chromosomes with the longest inserts of alien chromatin are found in a disomic state, whereby the process of pairing in meiosis should proceed without disturbances, ensuring the formation of functional gametes. Meanwhile, according to C-banding, three of the studied lines (25–2, 34–1 and 34–2) and according to microsatellite analysis, most lines (lines 19, 20–1, 25–2, 34–1) contain translocations involving the long arm of chromosome 5B (Table 1), where a *Ph1* locus, the main regulator of chromosome synapsis, is localized. Depending on the location of a translocation breakpoint in 5BL, those translocations could lead to the complete *Ph1* locus removal, or the changes in its expression. In this regard, a comparative study of chromosome behavior at different stages of microsporogenesis in hybrid lines and their parental forms was of great interest.

Analysis of metaphase I revealed a high level of bivalent chromosome pairing in all F_10_ lines (Table 2). A slight weakening of synapses in the line 25–2, containing pairs of aberrant chromosomes T3GS-3BS.3BL and Т5GS.5GL-5BL, is most likely to be a result of structural 3B chromosome transformations, the short arm of which contains a gene that prevents asinapsis (in the case of *Ph1* locus dysfunction, the consequences would be more radical). As shown earlier [23], the absence of 3B chromosome or its short arm in the soft wheat karyotype leads to the partial asynapsis of homologues that was observed in our material. The greatest number of PMC with disorders at metaphase I (73.3%) and the maximum number of univalents (6–3.3% of PMC) was found in the 34–2 line containing a pair of aberrant Т5ВS.5ВL-5GL chromosomes that is, of course, caused by the structural rearrangements of chromosome 5B and changes in the *Рh1* locus expression. At the same time, in the second line (34–1) from the same cross combinations, a negative effect of the similar introgression of chromatin 5G-chromosome is less pronounced: the number of PMC with disorders is 26.6%, which is, however, 3–4 times higher than for the lines with translocations not involving 5B chromosome. These differences may be due to the different length of an alien fragment.

The meiotic index of F_10_ lines isolated from the Rassvet×*T. kiharae* cross combinations and *T. kiharae*×Saratovskaya 29 was 6–9% lower than that of parental wheat varieties, but exceeded the 90% level even in line 25–2, for which the weakening of synapsis in metaphase I was observed. Lines 34–1 and 34–2 from the *T. kiharae*×Festivalnaya cross combination are characterized by the lowest meiotic index (77.0 and 55.83%, respectively). These results are in good agreement with the data of chromosome behavior of the lines at metaphase I and confirm a negative impact of the structural rearrangements identified in 5B chromosomes. The impact of the genetic background of the parental wheat variety Festivalnaya with the lowest meiotic index among the varieties used for hybridization should not be excluded either. (Table 3).

## Conclusion

The study results indicate that in the course of hybrid karyotypes’ stabilization, the introgression of the *T. kiharae* genetic material into the *T. aestivum* genome occurs both in the form of short fragments detected by molecular markers only and in the form of whole chromosome arms (intergenomic substitutions) and their fragments (centric and terminal translocations). The necessary conditions for the preservation of alien substitutions and translocations in the common wheat genome are their transition to the disomic state, as well as the participation in their formation chromosomes of orthologous genomes. Under these conditions, there are no significant disturbances in the process of gamete formation, which ensures the successful reproduction of introgression lines in a number of generations. The exception is introgressions affecting the functioning of main genes for chromosome synapsis. This results in a significant decrease of the meiotic index and negatively affects the productivity of plants.

## Methods

### Plant materials

In this study, we used the F_10_ lines obtained from three cross combinations of common wheat varieties with synthetic wheat *T. kiharae*: line 31 (Rassvet×*T.kiharae*); lines 19, 20–1, 25–2 (*T. kiharae*×Saratovskaya 29); lines 28, 34–1, 34–2 (*T. kiharae*×Festivalnaya). The lines were developed as a result of self-pollination of F_1_ hybrids and subsequent generations and selected for molecular cytogenetic studies based on the assessment of inheritance of morphological traits and productivity in generations F_2_ – F_9_.

Initial parental bread wheat cultivars (Rassvet, Festivalnaya) were received from the National Bank of Plant Genetic Resources of the Republic of Belarus. Seeds of bread wheat cultivar Saratovskaya 29 (K-40599) and *T. kiharae* (K-47897) were obtained from the National Genebank of Russian Federation (VIR, Federal Research Center N.I. Vavilov All-Russian Institute of Plant Genetic Resources, St. Petersburg; http://db.vir.nw.ru/virdb/maindb). *T. aestivum* x *T. kiharae* introgression line were developed by ecological genetics and biotechnology lab at the Institute of Genetics and Cytology, NAS of Belarus. All plant materials were deposited in the Institute of Genetics and Cytology, NAS of Belarus, Minsk.

### C-banding

Cytological preparation and C-banding were performed according to Badaeva et al. [24]. The seeds were germinated in Petri dishes at 26 °C. Seedlings with roots ranging in length from 0.5 to 1.5 cm were placed for 2 h in the 0.2% solution of colchicine (Fluka) at 26 °C. After that, the roots were washed with cold distilled water, cut off and transferred to ice water for an hour. Acetic acid (45%) was used as a fixator; duration of fixation – 4 h at 2–4 °C. Acetic acid washing was carried out six times in cold distilled water, for 10 min in each case. After washing, the roots were placed in 0.2 N HCl at 4 °C for 15 min. Hydrolysis was carried out in 0.2 N HCL at 60 °C for 5 min. After that, the roots were transferred to ice water. The roots were washed with cold distilled water (six times) for 5 min each. After that, the tips of the roots were cut off and placed in the 0.2% aqueous solution of cellulysin (100,000 cu, “Calbiochem”) at 26 °C for 14 h. The enzyme was washed with cold distilled water. The tips of the roots were macerated for 3 min in 45% acetic acid at room temperature on the slide and then were crushed. The cover glass was removed after slide freezing in liquid nitrogen. The slides were placed in 96° alcohol and stored at 4 °C. The dried slides were treated with the saturated solution of barium hydroxide at room temperature for 6 min. After alkali, the preparations were rinsed first in 1 N HCI for no more than 15 s, then thoroughly washed in running water and dried with hot air. After that, the slides were incubated in the solution of 2 × SSC (0.3 M NaCl + 0.03 M Na citrate, pH = 7.0) at 60 °C for an hour, then washed with running water for 15 min and dried.

Staining was performed with the 10% Giemsa solution (“Merck”) at 0.125 M Tris-HCl buffer (“Serva”) pH = 6.8. The dye concentration and staining time were selected experimentally, focusing on the optimal staining time of 15–30 min. The slides were washed off with a stream of running water and then the slides were well dried with cold air and placed in xylene for dehydration. The prepared slides were placed in antelan (“Serva”).

Identification of individual chromosomes of A-, B-, D-, A^t^- and G-genomes was carried out according to the ideogramme of the differentially stained chromosomes developed by Badaeva et al. [5, 25]. Stained slides were analyzed using Amplival microscope (Carl Zeiss, Jena). Selected metaphase plates were photographed using the Leica DC 300 digital video camera. Processing of the obtained images was carried out using graphics editor Adobe Photoshop 2017.

### SSR-analysis

Genomic DNA was isolated from 5 to 7-day-old seedlings as described in Skolotneva et al. [26]. Genotyping of lines and parent varieties was performed using SSR markers (WMC, GSM, and GSM) mapped in the genome of hexaploid wheat [27, 28]. The polymerase chain reaction (PCR) was carried out as described in Röder et al. [28]. Separation of PCR fragments was performed on an ABI PRISM 3100 automatic sequencer (Applied Biosystems, USA). The size of fragments was calculated using the computer program ABI GeneScan (version 2.1) developed by Applied Biosystems. The program GenAlEx M. 6.5 [29] was used for the statistic processing of the results of SSR analysis.

### Microsporogenesis

Microsporogenesis was studied on temporary squashed preparations. Spikes were cut before leaving the leaf sheath and fixed in the ethanol-acetic mixture (3:1). A day after fixation, the material was transferred to 70% ethyl alcohol, where it was stored before analysis at t = + 2–4 °C. Acetoorcein (2%) was used as a dye. For each cross combination and initial forms, 30 plates of metaphase I and 50–80 plates of the following stages of meiosis (anaphase I and II, metaphase II, tetrads) were analyzed. The slides were analyzed on the microscope Amplival (Carl Zeiss, Yena) with Apochromate lens 100x aperture 1.32 MI. Statistical data analysis was carried out using Microsoft Excel.

## Supplementary information

**Additional file 1 Table S1.** The length of the amplification fragments (bp) of SSR markers in parental wheat varieties and introgression lines.

## Data Availability

Authors can confirm that all relevant data are included in the article and/or its supplementary information files. Any material used and/or generated in this study are available upon reasonable request, from the corresponding author.

## Abbreviations

C-banding: Centromere banding stain
DNA: Deoxyribonucleic acid
PCR: Polymerase chain reaction
PMC: Pollen mother cells
SSR: Simple sequence repeats
